# Tumeur pseudo papillaire et solide du pancréas

**DOI:** 10.11604/pamj.2018.31.212.11394

**Published:** 2018-11-28

**Authors:** Houcine Maghrebi, Amin Makni, Rami Rehaim, Anis Haddad, Wael Rebai, Mouna Ayadi, Amine Daghfous, Fadhel Fteriche, Faouzi Chebbi, Rachid Ksantini, Mohamed Jouini, Montasser Kacem, Zoubeir Ben Safta

**Affiliations:** 1 Service de Chirurgie A, Hôpital La Rabta, Tunis, Tunisie; 2 Service d’Oncologie Médicale, Hôpital Salah Azaiez, Tunis, Tunisie

**Keywords:** Pancréas, tumeur pseudopapillaire et solide, résection chirurgicale, Pancreas, solid pseudo-papillary tumor of the pancreas, surgical resection

## Abstract

Les tumeurs pseudopapillaires et solides du pancréas (TPPS) sont des tumeurs épithéliales rares. Dans la plupart des cas, il s’agit de tumeurs survenant chez la femme jeune dans la deuxième ou la troisième décennie de la vie. La survie après résection primaire approche 90% à 5 ans. Nous rapportons le cas d’une jeune patiente de la vingtaine qui présente une tumeur pseudopapillaire et solide du pancréas découverte devant des douleurs abdominales sans perturbations des bilans biologiques. La tomodensitométrie (TDM), l'imagerie par résonance magnétique (IRM) et l'échographie endoscopique ont révélé une masse bien limitée se développant au dépend de l'isthme pancréatique. L'exérèse complète de la tumeur a été réalisée. L’examen anatomopathologique confirmait le diagnostic de tumeur pseudopapillaire et solide du pancréas. En conclusion, les tumeurs pseudopapillaires et solides du pancréas doivent être évoquées comme un des diagnostics différentiels de toute masse pancréatique en particulier chez les jeunes femmes. L'exérèse chirurgicale procure un bon pronostic.

## Abstract (en)

Solid pseudo-papillary tumors of the pancreas (SPTP) are rare epithelial tumors. In most cases, they occur in young woman in the second or the third decades of life. Survival after primary resection is approximatively 90% at 5 years. We report the case of a 20-year old female patient with solid pseudo-papillary tumor of the pancreas detected given the onset of abdominal pain without laboratory tests’ disturbances. CT scan, MRI and endoscopic ultrasound showed well-defined mass in the pancreatic isthmus. Complete tumor resection was performed. Anatomo-pathological examination confirmed the diagnosis of solid pseudo-papillary tumor of the pancreas. Solid pseudo-papillary tumor of the pancreas should be included in the differential diagnosis of any pancreatic mass, in particular among young women. Surgical resection is associated with a good prognosis.

## Introduction

Les tumeurs pseudopapillaires et solides du pancréas (TPPS) sont des tumeurs rares d’étiopathogénie encore incertaine. Elle touche le plus souvent la femme jeune. Le traitement est chirurgical et le pronostic est relativement favorable, avec un faible potentiel de malignité. Nous rapportons une nouvelle observation de tumeurs pseudopapillaires et solides du pancréas à travers laquelle nous insisterons sur les difficultés diagnostiques et thérapeutiques de cette entité rare.

## Patient et observation

**Figure 1:**
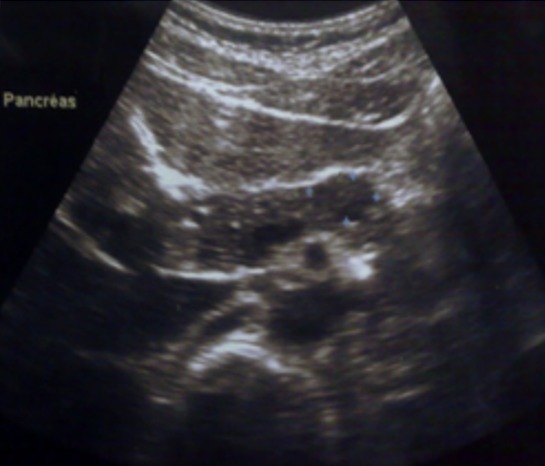
échographie abdominale montrant un nodule tissulaire hypoéchogène du corps du pancréas

**Figure 2:**
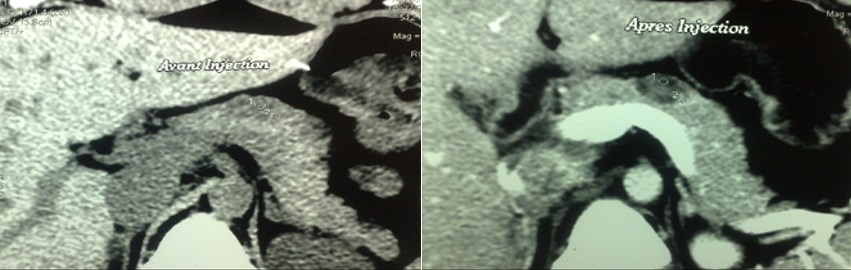
TDM abdominale montrant le nodule hypodense qui se rehausse après injection d’iode

**Figure 3:**
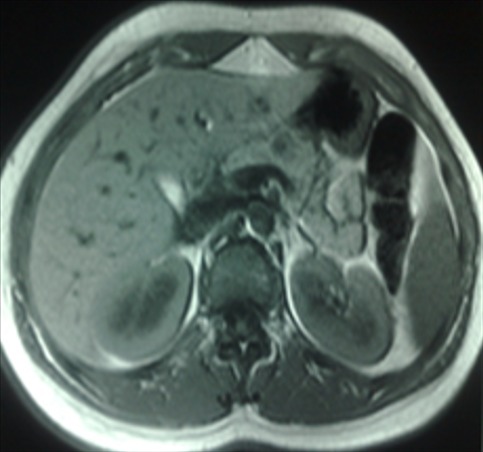
IRM montrant un nodule du corps du pancréas, en hypersignal T2, hyposignal T1

**Figure 4:**
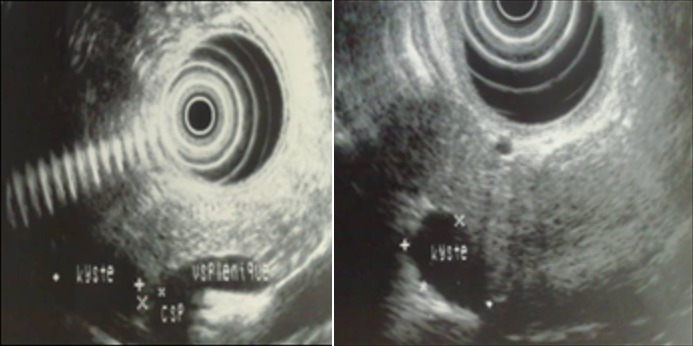
échoendoscopie montrant le nodule

Une patiente de la vingtaine, sans antécédents pathologiques particuliers, était admise pour exploration d’épigastralgies évoluant depuis 6 mois. L’examen physique était normal. La biologie était sans anomalie avec en particulier des marqueurs tumoraux (antigène carcino embryonnaire et CA 19-9) normaux. L’échographie abdominale (Figure 1) montrait un nodule tissulaire hypoéchogène du corps pancréatique de 20*10mm sans autre lésion associée. La tomodensitométrie abdominale (Figure 2) trouvait une lésion nodulaire hypodense grossièrement ovalaire de 21 mm de grand axe, intéressant la région isthmo-corporéale pancréatique. Cette lésion se rehaussait de façon hétérogène après injection d’iode, sans anomalie péri pancréatique décelable. L’imagerie par résonance magnétique (Figure 3) objectivait un nodule kystique du corps du pancréas, en hypersignal T2, hyposignal T1. Cette lésion présente des cloisons fines se rehaussant après injection de Gadolinium. Un complément d’exploration par échoendoscopie+biopsie (Figure 4) avait montré une lésion kystique du corps pancréatique dont la ponction a conclu à une tumeur pseudopapillaire du pancréas. L’indication d’exérèse était retenue et la patiente était alors opérée par laparotomie. L’exploration trouvait une lésion de 20mm à l’aplomb de l’isthme pancréatique. Il avait été réalisé une pancréatectomie centrale avec anastomose pancréatico-jéjunale. Les suites opératoires étaient simples. L’examen anatomopathologique confirmait le diagnostic de tumeur pseudopapillaire et solide du pancréas.

## Discussion

Les tumeurs pseudopapillaires et solides du pancréas représentent une des formes anatomopathologiques rares des lésions kystiques du pancréas. Elle ne représente qu’environ 2% de l’ensemble des tumeurs pancréatiques [1]. Depuis sa première description par Frantz en 1959, environs 450 cas ont été rapportés dans la littérature, essentiellement sous forme de cas isolés. L’âge moyen de découverte se situe dans la troisième décennie. Dans la plupart des cas, il s’agit de tumeurs survenant chez la femme jeune tel le cas de notre patiente [2, 3]. Cependant la plupart des séries de la littérature retrouvent une origine ethnique noire ou asiatique contrairement à notre patiente qui était de race blanche.

Macroscopiquement, il s’agit d’une masse solide délimitée par une capsule et associant des zones d’hémorragie, de nécrose et de calcifications. Elle peut atteindre aussi bien la tête, le corps ou la queue du pancréas, avec néanmoins une prédominance dans la région corporéo-caudale [4]. Sa pathogénie est encore mal élucidée. Il peut s’agir de facteurs hormonaux comme en témoigne la prédominance féminine et la présence de récepteurs hormonaux dans ces tumeurs [5]. Cependant d’autres auteurs ont suggéré une origine embryonnaire impliquant une cellule souche totipotente qui se différencierait secondairement vers une cellule pancréatique [6]. Les TPPS sont découvertes fortuitement lors d’un examen radiologique de routine, à l’occasion de douleurs abdominales comme en témoigne le cas de notre patiente qui se plaignait d’épigastralgies isolées [2]. Rarement, la tumeur est découverte à l’occasion d’une complication (hémorragie intratumorale ou rupture intrapéritonéale) ou devant des signes de compression digestifs ou biliaires.

Autrefois le diagnostic était rarement fait en préopératoire. Actuellement avec le progrès de l’imagerie, le diagnostic peut être orienté par le contexte clinique. L’aspect le plus évocateur est celui d’une tumeur bien limitée, arrondie ou ovalaire, peu vascularisée et de composition mixte, associant des zones solides et des zones kystiques donnant un aspect hétérogène en échographie. Les aspects observés en tomodensitométrie et en imagerie par résonance magnétique sont souvent plus évocateurs en montrant des foyers hémorragiques, une capsule fibreuse, ou un refoulement sans envahissement des organes de voisinage [4]. Comme le cas de notre patiente, le diagnostic différentiel se pose surtout avec les tumeurs neuroendocrines. Dans ces cas, le diagnostic peut être redressé par la ponction cytologie préopératoire malgré le risque de dissémination tumorale. Le seul traitement de cette tumeur est l’exérèse chirurgicale offrant un pronostic bien meilleur que celui des adénocarcinomes [7]. L'exérèse doit être la plus complète possible en évitant les résections trop conservatrices qui exposent au risque d'une récidive tumorale. En effet, après résection complète d’une tumeur non métastatique, la survie est de 97% à 5 ans. Cependant, le taux de récidive est non négligeable atteignant 10-15%.

## Conclusion

Les tumeurs pseudopapillaires du pancréas sont rares survenant particulièrement chez la femme jeune. Leur pronostic bien meilleur que celui des autres tumeurs pancréatiques justifie une attitude chirurgicale radicale, même pour les tumeurs métastatiques. La survie après résection complète est excellente mais associée à un taux de récidive non négligeable [8].

## Conflits d’intérêts

Les auteurs ne déclarent aucun conflit d’intérêts.
